# Nonproteinuric diabetic kidney disease

**DOI:** 10.1007/s10157-020-01881-0

**Published:** 2020-03-31

**Authors:** Masayuki Yamanouchi, Kengo Furuichi, Junichi Hoshino, Yoshifumi Ubara, Takashi Wada

**Affiliations:** 1 Nephrology Center, Toranomon Hospital, 2-2-2 Toranomon, Minato-ku, Tokyo, 105-8470 Japan; 2 Nephrology Center, Toranomon Hospital Kajigaya, 1-3-1 Kajigaya, Takatsu-ku, Kawasaki, Kanagawa, 213-8587 Japan; 3 Department of Nephrology and Laboratory Medicine, Faculty of Medicine, Institute of Medical, Pharmaceutical and Health Sciences, Graduate School of Medical Sciences, Kanazawa University, 13-1 Takaramachi, Kanazawa, Ishikawa, 920-0934 Japan; 4 Okinaka Memorial Institute for Medical Research, 2-2-2 Toranomon, Minato-ku, Tokyo, 105-8470 Japan; 5 Department of Nephrology, School of Medicine, Kanazawa Medical University, 1-1 Daigaku, Uchinada, Kahoku, Ishikawa, 920-0293 Japan

**Keywords:** Diabetic kidney disease, Diabetic nephropathy, Nonproteinuric diabetic kidney disease, End-stage kidney disease, Proteinuria

## Abstract

Proteinuria has been considered to be the hallmark of diabetic kidney disease and to precede renal function loss. However, it has become clear that a substantial proportion of patients either with type 1 diabetes or type 2 diabetes have renal function loss without proteinuria, known as nonproteinuric diabetic kidney disease. Despite increasing recognition of the prevalence of nonproteinuric diabetic kidney disease, data on this phenotype of diabetic kidney disease is sparse. This review describes ever known clinical and pathological manifestations, renal prognosis, and mortality in patient with nonproteinuric diabetic kidney disease.

## Introduction

Diabetic kidney disease is not just the most prevalent form of chronic kidney disease (CKD) but also it is the most leading cause of end-stage kidney disease (ESKD) worldwide [1–4]. Proteinuria, or macroalbuminuria, has been considered to be the clinical hallmark of diabetic kidney disease and an independent risk factor for ESKD [5, 6]. Patients with diabetic kidney disease are believed to develop proteinuria prior to renal function loss [7]. This classical belief, however, has been recently disputed by growing evidence that a substantial proportion of patients either with type 1 diabetes or type 2 diabetes have renal function loss in the absence of proteinuria, known as nonproteinuric diabetic kidney disease [8–13]. This phenotype of diabetic kidney disease suggests that there is a dissociation between renal function and level of albuminuria in patients with diabetes and highlight the need for broader understanding of renal function loss apart from those related to an increase in albuminuria. However, a limited number of studies have investigated nonproteinuric diabetic kidney disease.

In this review, we discuss ever known epidemiology, pathology, renal prognosis, and mortality of nonproteinuric diabetic kidney disease, comparing with those of proteinuric diabetic kidney disease. We also discuss potential mechanisms and perspectives of nonproteinuric diabetic kidney disease.

## Proteinuric diabetic kidney disease

### General concept of natural history of proteinuric diabetic kidney disease

**Fig. 1:**
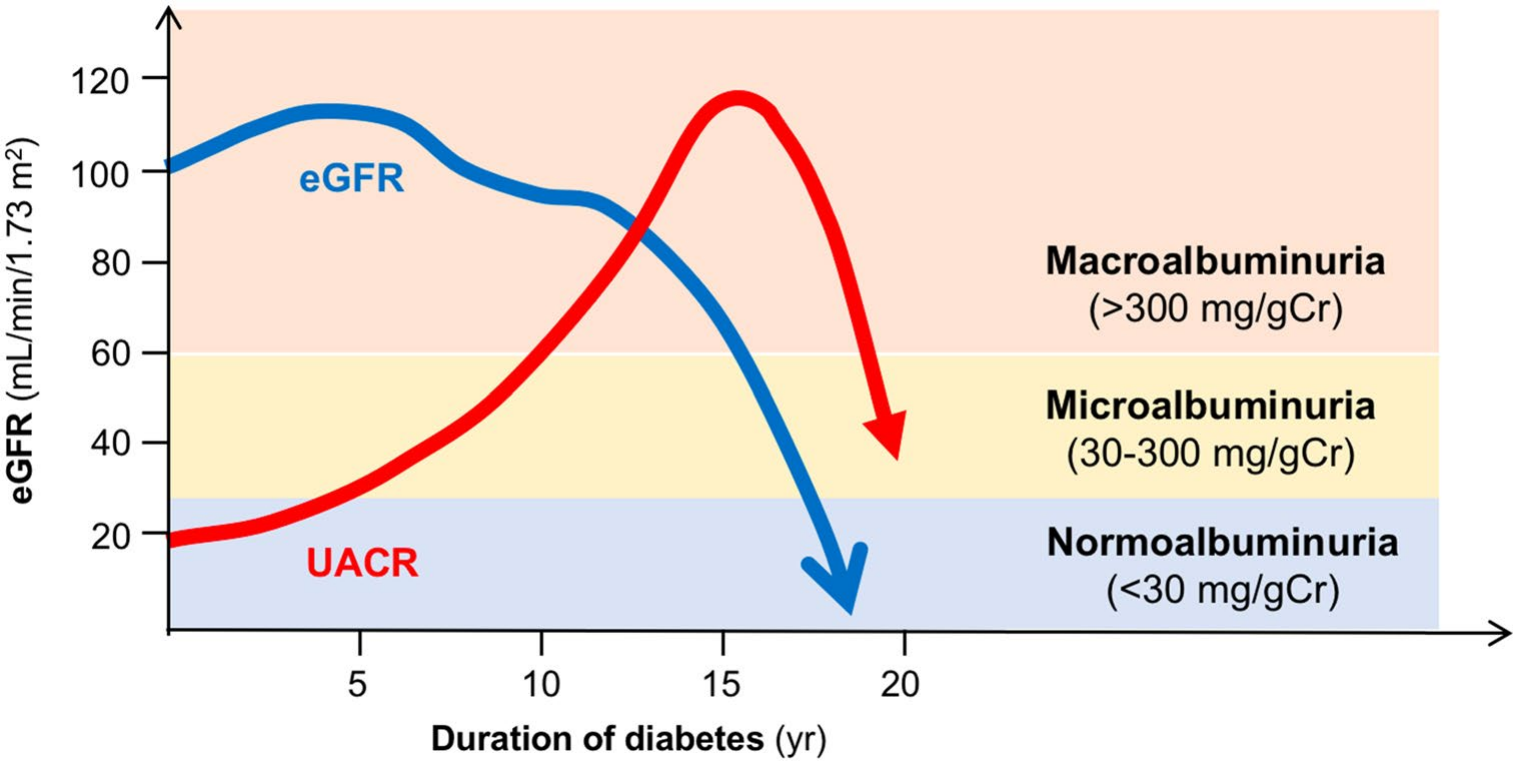
General concept of natural history of proteinuric diabetic kidney disease. Proteinuric diabetic kidney disease develops proteinuria prior to renal function loss. *eGFR* estimated glomerular filtration rate, *UACR* urine albumin to creatinine ratio

The general concept of natural history of proteinuric diabetic kidney disease has been formed by the observational studies mostly done in patients with diabetes before the current era of recommended multidisciplinary treatment including intensive glycemic control, tight blood pressure control, and renoprotective therapy such as renin-angiotensin system blockades, glucagon-like peptide-1 receptor agonists or sodium-glucose transport protein 2 inhibitors [7]. Patients with longstanding diabetes generally began with elevated glomerular filtration rate (known as glomerular hyperfiltration), and then developed proteinuria (urine protein to creatinine ratio (PCR) > 500 mg/g creatinine or macroalbuminuria; urine albumin to creatinine ratio (UACR) > 300 mg/g creatinine or mg/day) followed by microalbuminuria (UACR 30–300 mg/g creatinine or mg/day), which was once considered the onset of unidirectional process toward ESKD. Therefore, a renal function loss with an estimated glomerular filtration rate (eGFR) below 60 mL/min/1.73 m^2^ was also thought to occur after developing proteinuria (macroalbuminuria) (Fig. 1).

Of note, the term “diabetic nephropathy” was originally a pathological term that indicates a specific glomerulopathy including glomerular basement membrane thickening, nodular glomerular sclerosis, and mesangial expansion [14]. Historically, it has also been used as clinically diagnosed kidney disease in patients with longstanding diabetes and proteinuria, since patients with biopsy-proven diabetic nephropathy often accompany by proteinuria. However, the term “diabetic kidney disease” was first introduced in 1995 by Dr. Krolewski to indicate clinically diagnosed kidney disease in patients with diabetes [15]. Subsequently, the National Kidney Foundation adopted the term “diabetic kidney disease” in 2007 in its guidelines and clinical practice recommendations for the diagnosis and management of patients with diabetes and CKD [16]. The guidelines defined the term “diabetic kidney disease” a presumptive diagnosis of kidney disease caused by diabetes, and described “diabetic nephropathy” should be replaced by the term “diabetic kidney disease” for making easier to communicate among patients, caregivers, and policy makers. Since then, “diabetic kidney disease” has been used to include in the definition other than glomerulopathy or proteinuria typically seen in “diabetic nephropathy”. Notably, the guideline also described that “diabetic glomerulopathy” should be reserved for biopsy-proven kidney disease caused by diabetes. In addition, the Japanese Society of Pathology and the Japanese Society of Nephrology preserve the term “diabetic nephropathy” for biopsy-proven kidney disease caused by diabetes [17].

### Pathology of proteinuric diabetic kidney disease

As same with the natural history of proteinuric diabetic kidney disease, pathological lesions often seen in diabetes has been accumulated primarily in patients with diabetes before the contemporary era of multimodality therapy [14]. The early studies of morphological changes in poorly controlled diabetes reveal that specific lesions include diffuse lesions characterized by glomerular basement thickening and mesangial expansion, nodular lesions characterized by nodular glomerular sclerosis (known as Kimmelstiel-Wilson nodule), and hyalinosis lesions characterized by exudative/insudative lesion and fibrin cap. Especially, nodular glomerular sclerosis was considered the hallmark of proteinuric diabetic kidney disease observed in patients with longstanding diabetes and renal function loss.

Nowadays kidney biopsy is rarely performed in patients with diabetes unless they are suspected to have either superimposed non-diabetic kidney disease or de novo non-diabetic kidney disease. Particularly patients without proteinuria or albuminuria are rarely performed biopsy. However, a few previous biopsy-based studies revealed that patients without proteinuria have myriad of histological findings, suggesting that diabetic kidney disease is not only clinically but also pathologically heterogeneous [18–21].

## Nonproteinuric diabetic kidney disease

### General concept of nonproteinuric diabetic kidney disease

**Fig. 2:**
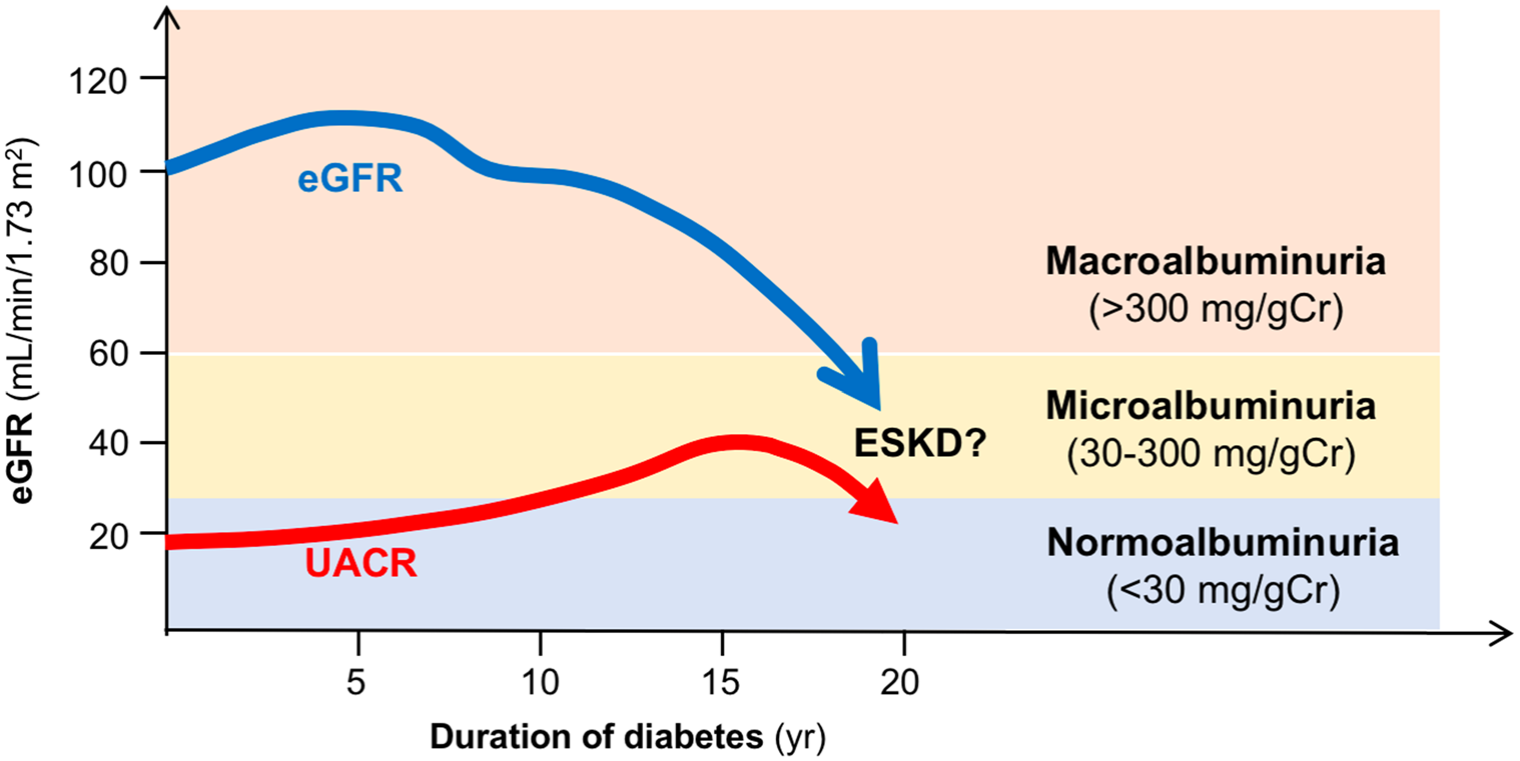
General concept of natural history of nonproteinuric diabetic kidney disease. Nonproteinuric diabetic kidney disease does not always develop proteinuria prior to renal function loss. In addition, it remains unclear whether nonproteinuric patients finally develop proteinuria and progress to ESKD. *eGFR* estimated glomerular filtration rate, *UACR* urine albumin to creatinine ratio

As seen above, proteinuric diabetic kidney disease is characterized by progressive renal decline with proteinuria seen mainly in patients with longstanding diabetes but without intensive treatment. However, growing evidence indicates that a substantial proportion of patients either with type 1 diabetes or type 2 diabetes have renal function loss without overt proteinuria, or have renal function loss even with normoalbuminuria [8–13] (Fig. 2). Although it is unclear whether this phenotype of diabetic kidney disease is due to an increase of elderly diabetic patients, or an increase of multidisciplinary treatment including renoprotective agents in general use, nonproteinuric diabetic patients with renal function loss (nonproteinuric diabetic kidney disease; defined as having an eGFR < 60 mL/min/1.73 m^2^ and UACR ≤ 300 mg/g creatinine) has come to the fore. Now current reports showed that the prevalence of nonproteinuric diabetic kidney disease are around 20% among patients with type 1 diabetes and around 40% among patients with type 2 diabetes, suggesting that diabetic kidney disease is now known to be clinically heterogeneous [22, 23]. Despite increasing recognition of the prevalence of nonproteinuric diabetic kidney disease, clinical pictures, pathological characteristics, renal prognosis, and mortality among nonproteinuric diabetic kidney disease have not fully investigated.

### Characteristics of nonproteinuric diabetic kidney disease

Cross-sectional studies report that clinical factors associated with nonproteinuric diabetic kidney disease include female gender, hypertension, smoking, hyperglycemia, no evidence of microangiopathy (represented as diabetic retinopathy), and the use of renin-angiotensin system blockade [8–13]. However, the clinical pictures of patients with nonproteinuric diabetic kidney disease were inconsistent across these previous reports.

These inconsistent findings may be due to the fact that diabetic kidney disease is clinically diagnosed. Patients with diabetes rarely undergo kidney biopsy and therefore patients clinically diagnosed with diabetic kidney disease may have non-diabetic kidney disease other than diabetic kidney disease. It is also possible that clinical characteristics can be changed depending on the timing of the diagnosis of diabetic kidney disease; for example, clinical characteristics in patients aged 35 may be different from those in patients aged 65, because aging impacts on duration of diabetes, renal function, and so on. We therefore investigated a propensity score matched nationwide cohort of biopsy-proven diabetic kidney disease to address these imbalances between nonproteinuric diabetic kidney disease and proteinuric diabetic kidney disease [24]. Of them, 88 were nonproteinuric diabetic kidney disease (UACR ≤ 300 mg/g creatinine) and 438 were proteinuric diabetic kidney disease (UACR > 300 mg/g creatinine). To fairly compare clinical features, we used a propensity score matching groups of 82 nonproteinuric diabetic kidney disease and 164 proteinuric diabetic kidney disease. We have shown that patients with nonproteinuric diabetic kidney disease have better-controlled blood pressure and lipid profiles, compared to patients with proteinuric diabetic kidney disease (Table 1). Of note, the nonproteinuric diabetic kidney disease group was less prescribed renin-angiotensin system blockades (48%), compared to the proteinuric diabetic kidney disease group (69%).

**Table 1:** Clinical characteristics among nonproteinuric and proteinuric diabetic kidney disease

Clinical characteristics at biopsy	Propensity Matched Cohort		
	Nonproteinurics (*n* = 82)	Proteinurics (*n* = 164)	*p *value
Age (year)	63 (56, 67)	64 (56, 70)	0.52
Male (%)	66	68	0.68
BMI (kg/m^2^)	23 (21, 25)	24 (22, 26)	0.098
Diabetes duration (year)	12 (8, 18)	13 (8, 21)	0.45
Retinopathy (%)	62	69	0.44
Smoking (%)	63	61	0.90
RAAS (%)	48	69	0.015
Glucose-lowering agents (%)	93	90	0.57
Statin (%)	31	20	0.21
Systolic blood pressure (mmHg)	130 (120, 145)	146 (134, 162)	< 0.001
Diastolic blood pressure (mmHg)	75 (68, 80)	80 (70, 90)	0.009
Hemoglobin A1c (%)	7.2 (6.5, 9.0)	6.9 (6.0, 8.3)	0.033
Total cholesterol (mmol/L)	5.0 (3.9, 5.8)	5.4 (4.6, 6.4)	0.002
Triglycerides (mmol/L)	1.5 (1.1, 2.2)	1.7 (1.2, 2.4)	0.21
LDL-C (mmol/L)	2.8 (2.1, 3.4)	3.3 (2.6, 4.1)	0.033
Uric acid (mg/dL)	6.8 (5.9, 7.5)	6.5 (5.7, 7.8)	0.90
eGFR (mL/min/1.73 m^2^)	45 (33, 54)	44 (29, 50)	0.12
UACR (mg/g creatinine)	100 (30, 180)	2100 (1140, 3570)	
Albuminuria status (*n*)			
Normoalbuminuria	19	0	
Microalbuminuria	63	0	
Macroalbuminuria	0	164	

Adapted from Yamanouchi et al. [24]. Copyright 2019 by the American Diabetes Association

Data are expressed as the mean (standard deviation), median (25th, 75th percentiles), or percentage

*BMI* body mass index, *Retinopathy* diabetic retinopathy, *RAAS* renin–angiotensin–aldosterone system blockade, *sBP* systolic blood pressure, *dBP* diastolic blood pressure, *LDL-C* low-density-lipoprotein cholesterol, *HDL-C* high-density-lipoprotein cholesterol, *eGFR* estimated glomerular filtration rate, *UACR* urine albumin to creatinine ratio. Albuminuria status, normoalbuminuria: UACR < 30 mg/g; microalbuminuria: UACR 30–299 mg/g; macroalbuminuria: UACR > 300 mg/g

### Pathology of nonproteinuric diabetic kidney disease

A limited number of studies have investigated morphological features of nonproteinuric diabetic kidney disease. However, findings from these biopsy-based studies vary depending on the era, or depending on the type of diabetes. Back in the early 2000s, a biopsy study done in patients with type 1 diabetes showed that typical glomerular features associated with diabetic nephropathy (diabetic glomerulopathy) are often observed in nonproteinuric diabetic kidney disease, although this study did not deeply investigate interstitial and arterial features [18]. Meanwhile, after 2010, biopsy studies in patients with type 2 diabetes showed that typical glomerular features associated with diabetic nephropathy (diabetic glomerulopathy) are less frequently observed in nonproteinuric diabetic kidney disease, although findings of interstitial and arterial features changed depending on the study [19–21].

**Table 2:** Pathological characteristics among nonproteinuric and proteinuric diabetic kidney disease

Pathological characteristics at biopsy	Propensity matched cohort		
	Nonproteinurics (*n* = 82)	Proteinurics (*n* = 164)	*p* value
Fioretto classification (%)			< 0.001
CI	62	17	
CII	20	66	
CIII	18	17	
Tervaert (RPS) classification (%)			< 0.001
I	31	4	
IIa	22	14	
IIb	10	20	
III	25	52	
IV	2	10	
Japanese classification			
Glomerular lesions			
GS (%)	16 (6, 37)	33 (17, 44)	< 0.001
Diffuse lesion (%)			< 0.001
0	16	1	
1	43	17	
2	17	29	
3	24	53	
GBM doubling (%)			0.001
0	66	23	
1	17	41	
2	8	23	
3	9	13	
Exudative lesion (%)	24	61	< 0.001
Nodular lesion (%)	22	54	< 0.001
Mesangiolysis (%)	19	49	< 0.001
Polar vasculosis (%)	54	73	0.014
Glomerulomegaly (%)	26	37	0.13
Interstitial lesions			
IFTA (%)			< 0.001
0	11	2	
1	53	24	
2	23	37	
3	13	37	
Inflammation (%)			0.021
0	15	4	
1	62	64	
2	18	22	
3	5	10	
Vascular lesions			
Arteriolar hyalinosis (%)			0.002
0	15	4	
1	23	16	
2	29	48	
3	33	32	
Arteriosclerosis (%)			0.002
0	16	5	
1	35	51	
2	47	44	
3	2	0	

Adapted from Yamanouchi et al. [24]. Copyright 2019 by the American Diabetes Association

Data are expressed as the mean (standard deviation), median (25th, 75th percentiles), or percentage

Fioretto Classification; CI, normal or near normal renal structure; CII, typical diabetic kidney disease; CIII, A typical patterns of renal injury; Tervaert (RPS) classification, Renal Pathology Society diabetic kidney disease classification; I; Mild or nonspecific light microscopy changes and electron microscopy-proven glomerular membrane thickening; IIa; Mild mesangial expansion; IIb, Severe mesangial expansion; III; Nodular sclerosis (Kimmelstiel-Wilson lesion); IV, Advanced diabetic glomerulosclerosis; GS, percentage of glomerulosclerosis defined as the number of global or segmental sclerosis glomeruli per total glomeruli; GBM, glomerular basement membrane; IFTA, interstitial fibrosis and tubular atrophy

**Fig. 3:**
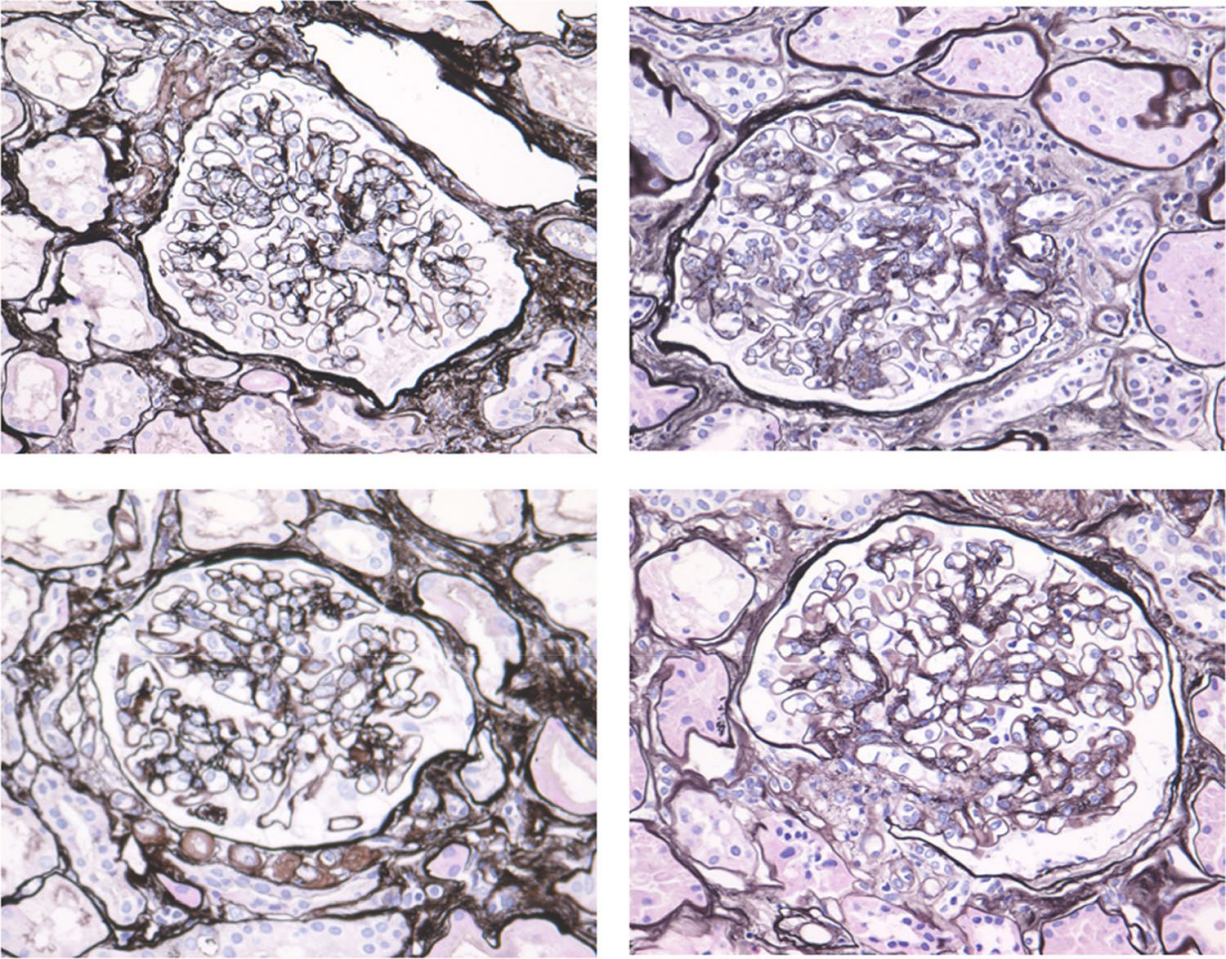
Pathology of nonproteinuric diabetic kidney disease. Majority of patients with nonproteinuric diabetic kidney disease have minor changes in glomerular histology

These inconsistent findings may be due to a small number of study population. They may also arise from the timing of the biopsy; for example, clinical characteristics in patients with eGFR 50 mL/min/1.73 m^2^ may be different from those in patients with the same backgrounds but with eGFR 25 mL/min/1.73 m^2^; or age and duration of diabetes may affect pathological findings. Again, we therefore investigated a propensity score matched nationwide cohort of biopsy-proven diabetic kidney disease to address these imbalances between nonproteinuric diabetic kidney disease and proteinuric diabetic kidney disease [24]. We have shown that patients with nonproteinuric diabetic kidney disease have fewer of typical morphological features, not only in glomerulus but also in interstitium and arterioles, associated with diabetic nephropathy (diabetic glomerulopathy) (Table 2 and Fig. 3). For example, the prevalence of glomerular nodular lesions was 22% in nonproteinuric diabetic kidney disease and 54% in proteinuric diabetic kidney disease.

### Renal prognosis and mortality of nonproteinuric diabetic kidney disease

A couple of contemporary studies showed that those with nonproteinuric diabetic kidney disease carry a lower risk of progression of renal function and death. A study from the Steno Diabetes Center analyzing eGFR trajectories of 935 patients with type 1 diabetes and 1984 patients with type 2 diabetes for up to 16 years after developing CKD stage 3 (eGFR < 60 mL/min/1.73 m^2^) has shown that the trajectories of nonproteinuric diabetic kidney disease has less progressive declining trajectories of proteinuric diabetic kidney disease [25]. The annual change in eGFR decline for normoalbuminuria, microalbuminuria, and macroalbuminuria following eGFR < 60 mL/min/1.73 m^2^ were 1.9, 2.3, and 3.3 mL/min/1.73 m^2^ in type 1 diabetes and 1.9, 2.1, and 3.0 mL/min/1.73 m^2^ in type 2 diabetes, respectively. Other study from Australia has shown that annual change in eGFR decline for normoalbuminuria and albuminuria were 0.6 and 1.75 mL/min/1.73 m^2^, respectively [26]. The hazard ratio for death was lower for nonalbuminurics, than for albuminurics (1.42 vs. 2.38). In addition, a study from Japan has also shown that the 5-year CKD progression-free survival were 86.6% (95% CI 72.5–93.8) for the nonproteinuric diabetic kidney disease group and 30.3% (95% CI 22.4–38.6) for the proteinuric diabetic kidney disease group (log-rank test, *p* < 0.001) [24]. The lower renal risk was consistent across all subgroup analysis. The all-cause mortality was also lower in the nonproteinuric group (log-rank test, *p* < 0.001).

## Conclusion and perspectives

Diabetic kidney disease has been clinically diagnosed based on the traditional belief that patients with diabetic kidney disease present proteinuria followed by renal function loss, and glomerular nodular lesions are observed if these patients underwent kidney biopsy. However, a number of clinical and pathological studies of diabetic kidney disease we have reviewed in this compendium have uncovered the evidence that diabetic kidney disease is clinically and pathologically heterogeneous, suggesting that there may be various phenotypes of diabetic kidney disease. One of these phenotypes is nonproteinuric diabetic kidney disease characterized by renal function loss (eGFR < 60 mL/min/1.73 m^2^) in the absence of proteinuria (UACR ≤ 300 mg/g creatinine or mg/day). This phenotype of diabetic kidney disease suggests that there is a dissociation between renal function and level of albuminuria in patients with diabetes and highlight the need for broader understanding of renal function loss apart from those related to an increase in albuminuria.

A handful of clinical studies raised potential mechanisms of becoming nonproteinuric diabetic kidney disease. One possibility is an increase in elderly patients with diabetes. Senescence of the kidney occurs with aging, which may cause an eGFR below 60 mL/min/1.73 m^2^, even if the impact of diabetes on renal function is little. Additionally, elderly patients with diabetes often have increased underlying conditions such as hypertension, dyslipidemia, obesity, and hyperuricemia, all of which may cause renal function loss via arteriosclerosis, known as nephrosclerosis. Indeed, the primary pathological findings in nonproteinuric diabetic kidney disease are similar findings to hypertensive nephrosclerosis, characterized by glomerular sclerosis, interstitial fibrosis and tubular atrophy, and arteriosclerosis [19, 24, 27–29]. Another possibility is that patients with nonproteinuric diabetic kidney disease is mostly comprised of those who responded well to renin-angiotensin system blockades that results in nonproteinuria via protecting glomerulus. A meta-analysis of 28 cohorts including 693,816 patients (80% with diabetes) and 7461 ESKD events has shown that 30% reduction in albuminuria over 2 years was associated with around 20% risk reduction of ESKD, suggesting that regression of albuminuria may be a favorable prognostic indicator [30]. However, whether nonproteinuric patients finally develop proteinuria and progress to ESKD, despite of multifactorial therapy, is of great interest. Other possibility is macroangiopathy. A couple of studies report that the prevalence of diabetic retinopathy is lower in those with nonproteinuric diabetic kidney disease than those with proteinuric diabetic kidney disease, suggesting microangiopathy may not be the main pathogenic factor, rather past history of macrovascular disease such as cardiovascular disease may be a potential pathogenic factor in nonproteinuric diabetic kidney disease [23]. However, this mechanism seems doubtful from our results showing that there were no differences in prevalence of retinopathy and CVD events among nonproteinuric and proteinuric diabetic kidney disease [24, 31].

Although our study has shown that patients with nonproteinuric diabetic kidney disease carry a lower risk of progression of renal function loss, compared to those with proteinuric diabetic kidney disease, around 20% of those with nonproteinuric diabetic kidney disease experienced progression to advanced CKD or ESKD in 10 years [24]. Those who progressed to advanced CKD or ESKD had more severe interstitial fibrosis and tubular atrophy, compared to those who did not progress, suggesting that in the absence of proteinuria, tubular damage may play an important role in progression of CKD. However, whether those who progressed to advanced CKD finally develop proteinuria needs to be clarified. Data on albuminuria followed up to ESKD is scarce. A study from the Steno Diabetes Center reported that around 20% of diabetic patients in the absence of albuminuria never developed proteinuria before ESKD, suggesting that developing to proteinuria is not a prerequisite for ESKD [25]. This finding suggests that underlying pathogenesis may different among glomerulus and interstitium. However, this study included patients with clinical diagnosed diabetic kidney disease in single center and therefore it still remains to be elucidated whether nonproteinuric patients finally develop proteinuria and progress to ESKD in other cohorts. Exploration of biomarkers apart from level of albuminuria may elucidate a mechanism of progression of nonproteinuric diabetic kidney disease. For example, an analysis of a nationwide biopsy-based cohort in Japan with a thorough glomerular investigation showed that diffuse lesions, polar vasculosis and subendothelial space widening predict the prognosis of advanced CKD even in the absence of proteinuria [21]. Notably, the subendothelial space widening also provides prognostic value on predicting CVD events in patients with nonproteinuria. Other examples are that some studies report that potential mechanisms of progression of advanced CKD include inflammation markers such as TNF and Fas pathways, and tubular damage markers such as KIM-1, all of which are reported to be independent of level of albuminuria [32, 33].

Recent studies focused on nonproteinuric diabetic kidney disease have elucidated its clinical, pathological features, renal prognosis, and mortality. However, further studies are needed to fully comprehend its mechanism and retard its progression of CKD.
